# Quality criteria in MOOC: Comparative and proposed indicators

**DOI:** 10.1371/journal.pone.0278519

**Published:** 2022-12-05

**Authors:** Camino Ferreira, Ana R. Arias, Javier Vidal

**Affiliations:** 1 Department of Psychology, Sociology and Philosophy, University of León, León, Spain; 2 Department of General and Specific Didactics and Educational Theory, University of León, León, Spain; Hefei University of Technology, CHINA

## Abstract

The MOOC (Massive Open Online Course) offer revolution requires processes that can define their quality, especially due to the number of courses offered, as well as the high number of students enrolled in them. The objective of this study is to identify the main requirements and indicators of the MOOC following the ENQA (The European Association for Quality Assurance in Higher Education) considerations. To establish this system, the study has been carried out through the Delphi method with successive rounds of application from the systematic use of expert judgment. This method has been applied to achieve consensus on a set of requirements and indicators of 20 experts from eight different institutions in the field of application and development of MOOCs that assessed the indicators according to the quality criteria according to three aspects: relevance, feasibility, and comparability. Therefore, the outcome of this study is a system as a mechanism for the university to approve or disapprove a MOOC (checklist) and assess its quality.

## Introduction

The phenomenon of MOOCs (Massive Open Online Course) began in 2008 and rapidly gained momentum in the following years thanks to the main virtual education platforms [1], aimed at a lifelong learning audience of adults [2]. These include Coursera (founded by Stanford University professors), Udacity (which partners are Google and Microsoft among others) and edx (belonging to MIT and Harvard University), founded between 2011 and 2012 [3]. MOOCs are characterised, as their name suggests, by being courses with free (open) access, with no limit on the number of students participating (massive), with a design based on a collaborative and interactive participatory methodology with minimal intervention by the teacher, mainly through audiovisual materials available online [4]. In addition to these characteristics, MOOCs have a specific duration (start and end date) and include assessment systems, but not admission systems [5]. MOOCs are more appropriate for public elective courses and open courses to the society [6].

### Literature review

MOOCs are part of what is considered virtual education or e-learning. E-learning differs from conventional teaching in higher education in the type of degree that can be obtained, in the profile of the students (e-learning students usually work full time), in the teaching staff as counsellors or tutors who monitor and support the students, in the virtual technological infrastructure used and in the high degree of homogeneity of the teaching process (same materials, activities, same support system, etc.) [7]. The same applies if we differentiate between e-learning and MOOC, since not everything within e-learning can be considered as such (see Table 1). The European Association for Quality Assurance in Higher Education (ENQA) has recently published considerations for quality assurance of e-learning provision, defining e-learning terminology in which MOOCs are delimited as “online courses that are designed for large numbers of participants, often offered for free and without any entry qualifications. They are distinguished from Open Educational Resources (OERs) in that they offer a full course experience and content that is not usually free to reuse” [8]. MOOCs should also be distinguished from OER. These resources are considered by UNESCO as educational support materials characterised by the fact that they are freely accessible and can be reused, modified, and shared. They are therefore teaching-learning or research materials (e.g. interactive materials, course materials, course books, study books, videos, etc.) that are "open", i.e. available in any public medium, allowing their use, access, reformulation, reuse and redistribution [9].

**Table 1:** Characteristics of MOOCs and e-learning courses.

	MOOC	E-learning course
Admission	Massive (thousands)	Limited (tens to hundreds)
Access requirement	No (e-mail address)	Yes (selective admission or on-space-available basis)
Fee/Prices	Free	Payment of registration fee
Specific duration	Specified	Specified
Environment	Open	Closed
Support	Community, instructors, tutors, counsellors	Teaching staff
Instructor contact	Minimum (none expected)	Maximum (24-hour response)
Communication tools	Variety and optional, social networks	Debate forums (required and evaluated)
Sessions	Open automatically	Open at very specific moments
Content media	Video	Textbook
Emphasis	Learning process	Evaluation and accreditation
Platform	Focus on the dissemination of the activities	Focus on the interaction with lecturers
Evaluation system	Included (machine-scored; peer)	Included (instructor; machine-scored)
Certification	No (optional)	Always

Note. Own elaboration.

However, in many cases, "open" and "openness" are only referred to as general characteristics without a precise definition. According to Stracke, Open Education “covers and addresses all dimensions related to operational, legal and visionary aspects throughout the analysis, design, realization and evaluation of learning experiences to facilitate high quality education meeting the given situation, needs and objectives” [10]. With this definition in mind, according to Stracke, not only should access be open, but also the following dimensions should be open of openness: legal dimensions of openness (open availability, open licensing, and open access), operational dimensions of openness (open standards, open technologies, and open resources) and visionary dimensions of openness (open recognition, open innovations and open methodologies).

The certification of MOOC raises challenges and issues to be addressed, especially in pedagogical design, homogenisation and globalisation of culture, free of charge, strategies and positioning of companies, etc. [1]. Within these issues, there is also the role of the university, which must consider what these MOOCs represent within its strategic lines and within its Third Mission Activities. These activities refers to those that contribute to society in a meaningful way by means of Lifelong Learning, and also Technology Transfer and Innovation, and Social Engagement [11].

Despite the advantages that MOOCs offer, such as easy access, free tuition, a good supply of video material, excellent teacher presentation and a peer learning community; Johnston [12] identified some limitations:

- Lack of instructor-student feedback and instructor interaction.
- Reliance on machine-graded and peer-graded assessments.
- MOOC fail to meet many student expectations.
- Focus on low-level learning.

Given the number of MOOCs offered, the massive participation of students in them and the necessary institutional support and commitment to achieve high-quality courses on technology-based platforms, one of the main concerns and lines of research raised in several studies on these courses is the assessment of their quality and the development of indicators [5, 13, 14].

In some cases, this training offered by educational organisations does not guarantee the minimum quality compliance required by the students of the courses [4]. Aleman de la Garza [15] concludes that “the success of a MOOC cannot be evaluated without measuring results. Thus, institutions and consortia should establish indicators to focus efforts on improving their pedagogical quality”. This is why quality indicators can help improve course offerings and allow students to better choose courses according to their interests and needs, leading to greater student satisfaction [4]. It should be noted that the profile of students who use electronic educational resources in the learning process is characterised by being successful, motivated for learning activities and achieving better results, being motivated by the learning environment [16]. However, the gap between MOOC design and learner interaction with MOOCs has been studied, concluding that it is questionable whether MOOC course designers understand and meet the demands and needs of MOOC learners [17].

There are generic criteria for evaluating the e-learning environment that are also being used to evaluate the quality of MOOCs, but there is still a lack of homogenisation and unification of these criteria [18]. For all these reasons, there is a need for specific criteria to assess the quality of MOOCs, since not having them implies difficulty in systematically assessing the quality of these teaching and learning systems, and the absence of specific educational assessment criteria adapted to the characteristics of a MOOC [18]. This phenomenon seems to be one of the major drivers of change in higher education organisations, implying an innovation in higher education that requires benchmarking and quality assessment [14]. Furthermore, Grifoll et al. [7] reinforce that it is important that the evaluation of e-learning programmes should be of the same quality as non-distance learning degrees.

Several studies have established the dimensions in which evaluation tools should be configured. These include: learning design, communication-interaction, planning-management, accessibility levels and learning methodology [5]; organization and management, input, teaching process, output [19]; retention, certification of completion of the course, fulfilment of achievements, change in knowledge or attitude, student’s experience [20]; pedagogical and technical [21]. Other studies highlight the importance of including cognitive, social and metacognitive indicators [22] or the inclusion an indicators of that collects the commitment of an institution or a person to this new environment of digital education [14].

Based on this analysis, the aim of this study is to characterise MOOCs and identify the main requirements and indicators for their evaluation, defining a system of indicators that establishes which indicators are specific to MOOCs as opposed to those of training quality management in general and e-learning. This objective will allow future students of these courses to know which MOOCs are of the highest quality and will allow institutions to guarantee that the MOOCs offered are working properly. This study, using the Delphi method, allows consensus to be reached on the indicators for assessing the quality of MOOCs in e-learning.

## Method

To achieve the objective of this study, the characteristics that define a MOOC in the scientific literature and the indicators (internal quality assurance indicators] included in the ENQA document *Considerations for quality assurance of e-learning provision* [8] were used as a starting point. On the one hand, 11 indicators have been selected that refer to the requirements that a MOOC must have (checklist), i.e., those aspects that are essential for it to be considered a MOOC. On the other hand, from the 58 ENQA indicators, 31 have been selected, eliminating 27 because they are evaluation indicators at institutional level or because they are specific to e-learning in general and do not apply to MOOCs. This process, in which the selection and definition of indicators has been reviewed, has been carried out through consultation with MOOC experts. The indicators have been ordered according to their importance at the time a MOOC is carried out: before (design), during (methodologies, resources, and support) and after (evaluation, quality system and dissemination).

Once the indicators had been selected, they were modified by changing the term e-learning to MOOC. Subsequently, the Delphi method has been used for the expert validation of this set of indicators, with two successive rounds of application. This Delphi method is based on a double-round iterative process (the answers are anonymous) and its purpose is to obtain a degree of consensus or agreement among experts on the need for a proprietary system of MOOC indicators and their definition.

This method was applied by means of in-depth questionnaires with feedback via Google Forms and Excel by e-mail during the year 2021. The participation in the survey was completely voluntary and the responses obtained have been confidential, allowing opinions to be based on the ideas of the consultation, avoiding bias derived from the prestige or leadership of any member of the group. As this was an opinion survey, no ethics committee approval was required. A two-round iterative process was carried out, the second round based on the results of the previous consultation. In the second round, statistical information (frequencies) was included, feeding back the responses obtained in the first round (statistical response of the group). The indicators have been assessed according to three aspects: relevance, feasibility, and comparability.

- Relevance: Importance for the measurement of a MOOC
- Feasibility: Expected facility of obtaining the information
- Comparability: Possibility of making adequate comparisons between different MOOCs

**Fig 1:**
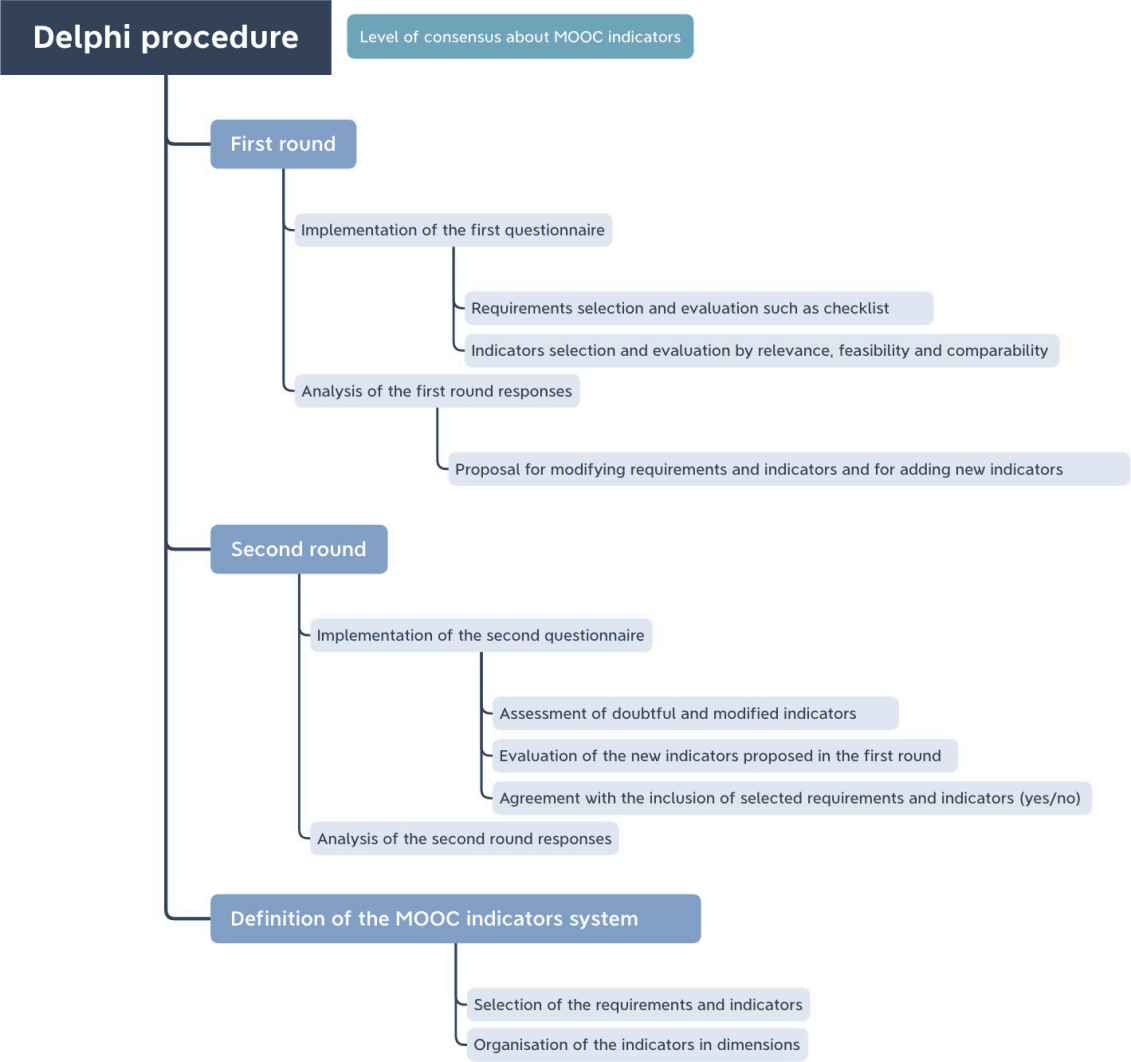
Delphi procedure. Note. Own elaboration.

The rounds and phases followed in the Delphi method were as follows (Fig 1):

The process has started with the 58 indicators defined by ENQA for e-learning [8]. The experts assessed the requirements and indicators for a MOOC to reach a consensus on the best indicators that characterise MOOCs. The degree of consensus determined in this study was greater than or equal to 70% on relevance and feasibility. The experts were also asked about additional indicators that they considered essential in the evaluation of MOOCs that were not included in the list.

The sample of experts participating in the Delphi consisted of 15 subjects (seven men and seven women). The sampling technique was non-probabilistic, purposive, or opinionated, selecting relevant subjects and experts with experience in the field of MOOC application and development. These experts come from different geographical regions: Spain (n = 8), France (n = 1), The United Kingdom (n = 3), Taiwan (n = 1) and Hungary (n = 1). The institutions to which the participating subjects belong are: The University of León, the University of Oviedo, the University Grenoble Alpes, the Open University, the National Kaohsiung Normal University, the University of Granada, the University of Alicante, and the Budapest Business School. Even though the sample consists of eight institutions, it was found that there were no significant differences between countries. All experts have experience in MOOC implementation, 50% have experience in MOOC design and 36% have experience in MOOC management. The results represent the synthesis of the expert group’s opinion, they do not analyse opinions at institutional level but at personal expert level.

## Results

The requirements to be met by MOOC to be considered as such will be assessed and indicators will be evaluated according to their relevance, feasibility, and comparability. The questionnaire is based on 11 MOOC’s requirements based on scientific literature and 31 selected indicators that has been defined by ENQA for e-learning [2018].

### Requirements

According to MOOC’s definitions of scientific literature, a MOOC is defined as having these 11 requirements. The expert indicated for each one if they consider it a requirement (see Table 2).

**Table 2:** Requirements results of the Delphi process.

Requirements	Results	Inclusion (1/0)	Percentage (2nd round)	Final proposal (yes/no)
RE11. The certification is optional	100%	1	100%	Yes
RE01. The MOOC admission is massive (thousands) and is not limited	93%	1	91%	Yes
RE03. The fee of the MOOC is free (there is no payment for registration)	93%	1	100%	Yes
RE08. The main content media of the MOOC are multimedia, interactive (not textbook, pdf)	86%	1	91%	Yes
RE09. The emphasis of the MOOC is focused on the learning process (not on evaluation)	86%	1	100%	Yes
RE10. The MOOC e-assessment system is included (machine-scored; peer)	79%	1	100%	Yes
RE04. The environment of the MOOC is open	71%	1	90%	Yes
RE06. There is a variety and optional communication tools, including social networks	71%	1	82%	Yes
RE02. There is no access requirement to the MOOC	64%	0	55%	No
RE05. The educational staff contact, and support are minimum or not expected	64%	0	55%	No
RE07. The session is opened automatically	50%	0	18%	No

The level of consensus determined is 70%, so the requirements with a score below this percentage have been rejected as such (RE02, RE05, RE07). Therefore, the remaining eight requirements (RE01, RE03, RE04, RE06, RE08, RE09, RE10, RE11) have been considered by the experts as requirements. There are no changes to the requirements set out, but there are comments in relation to them. In particular:

- RE06: There might be a variety of communication tools—or there might be only a few, I don’t think this is a requirement.
- RE08: A MOOC must comprise videos.
- On the cultural gap, the Taiwanese expert points out that in Taiwan *culture plays a key role to impact learners’ beliefs*. *Most learners still take the traditional learning method*. *MOOC courses are not the mainstream*.
- Finally, on the digital divide, it has also been indicated in relation to SR04 that *the environment of the MOOC is open to people who have access to a digital device with a stable internet connection and have some previous experience of independent learning*. *The content of the MOOC is not always open for people to reuse it outside the online environment*.

### Indicators

Considering the degree of expert consensus, 10 indicators have been eliminated for not reaching 70% consensus on the relevance criterion (IN011, IN27). Once analysed for relevance, if the indicator is relevant (70% or more), but the feasibility is less than 70% (IN01, IN09, IN10, IN11, IN12, IN18, IN19, IN20, IN27, IN29, IN30, IN31).

Some experts have proposed alternative wording to the following indicators:

- IN03. Student needs (including special educational needs if applicable) are considered when developing the learning model and the curricula design.
- IN07. Learning materials are relevant and are reviewed and updated periodically.
- IN08. The VLE supports the appropriate methods and tools that effectively support the achievement of the learning outcomes.

Following suggestions from the experts, the terminology has also been modified: *I would only change the wording of students for learners since a student is someone who is studying at a university*, *school etc*. *and a learner relates more to the profile of people learning in a MOOC*, *which includes also professionals or adult learners*. *The same would apply for teachers Vs MOOC educators/mentors etc*.

These indicators are supplemented by new indicators proposed by the experts (added in the second round):

- IN32. The MOOC fosters interactions between learners.
- IN33. Cultural factors are considered in the development of MOOC contents.
- IN34. There are clear and defined roles for the teaching staff involved in the MOOC, if applicable.

Indicators results of the Delphi process to be included is in Table 3 (n = 23) and those who are not in Table 4 (n = 11). For the design and formalisation of the indicators to be included, it is suggested that the elements established in the UNE 66175 standard for the implementation of indicator systems be considered: Indicator number (or code), dimension and name, objective, description, typology, calculation, representation, and source. It is suggested that all indicators are recorded at the nominal measurement level (yes/no calculation), represented by bar chart and annually.

**Table 3:** Indicators results of the Delphi process to be included.

Dimension	Indicator	Relevance	Feasibility	Comparability	Inclusion (1/0)	Percentage (2nd round)
Policy for quality assurance	IN28. The MOOC foresees an evaluation system for improvement that includes satisfaction surveys of stakeholders, especially learners (quality assurance system of the course itself)	86%	71%	57%	1	100%
Design and approval of programmes	IN02. People involved in designing/developing/evaluating MOOC programmes have expertise in academic and technical aspects	93%	86%	71%	1	91%
	IN03. Learner needs (including special educational needs if applicable) are considered when developing the learning model and the curricula design	86%	86%	71%	1	73%
Student-centred learning, teaching, and assessment	IN05. Teaching methodologies and learning activities are chosen with the aim of achieving learning outcomes	100%	100%	86%	1	100%
	IN06. Learning materials fit the pedagogical model and facilitate student learning	100%	86%	86%	1	100%
	IN14. E-assessment methods are fit for purpose, allowing students to demonstrate the extent to which the intended learning outcomes have been achieved	100%	71%	71%	1	100%
	IN16. Learners are aware of plagiarism rules	100%	71%	86%	1	100%
	IN07. Learning materials are relevant and are reviewed and updated periodically.	93%	86%	64%	1	100%
	IN15. Learners are clearly informed about the e-assessment	93%	79%	79%	1	100%
	IN08. The VLE provides the appropriate methods and tools that support effectively the achievement of the learning outcomes	93%	71%	79%	1	100%
	IN32. The MOOC fosters interactions between learners					82%
Student admission, progression, recognition, and certification	IN13. Learners are informed about the workload and pedagogical model of the MOOC programme	93%	79%	79%	1	91%
	IN17. Learners/prospective learners are informed about requirements concerning equipment, MOOC and digital skills, pre-knowledge and prerequisite subjects, and attendance	93%	71%	86%	1	82%
Teaching staff	IN21. Technological and pedagogical support services for educators are adequate, accessible, and timely	100%	79%	64%	1	100%
	IN04. There are coordination mechanisms for the educational staff involved, if applicable	86%	86%	79%	1	100%
	IN34. There are clear and defined roles for the educational staff involved in the MOOC, if applicable					82%
Learning resources and student support	IN22. The technical infrastructure ensures the accessibility of the MOOC programme by learners with special educational needs	100%	79%	86%	1	82%
	IN24. The MOOC guarantee the electronic security measures that guarantee standards of quality and information integrity and validity	86%	86%	79%	1	100%
Information management	IN25. The MOOC considers ethical norms and government policy with respect to data protection and the privacy of learners	100%	86%	93%	1	100%
	IN31. Collected data is used in order to evaluate MOOC programmes (e.g. comparative analysis of course design)	79%	64%	50%	0	82%
Public information	IN26. The MOOC publishes reliable, complete, and up-to-date information on MOOC (i.e. recognition of qualifications, learning objectives, credits, requirements, assessment methods, timelines, dates relevant for the programme)	93%	71%	64%	1	100%
	IN23. Technical requirements to enable the full and effective use of the system are clearly identified and published	86%	71%	86%	1	82%
Ongoing monitoring and periodic review of programmes	IN19. ICT and pedagogy developments are analysed and implemented when appropriate	93%	64%	71%	0	82%

**Table 4:** Indicators results of the Delphi process to NOT be included.

Dimension	Indicator	Relevance	Feasibility	Comparability	Inclusion (1/0)	Percentage (2nd round)
Policy for quality assurance	IN01. MOOC learning objectives is part of the overall strategy for the institution’s development as well as the policy for quality assurance	71%	64%	57%	0	18%
Design and approval of programmes	IN33. Cultural factors are considered in the development of MOOC contents.					64%
Student-centred learning, teaching, and assessment	IN20. The technical infrastructure is aligned with the teaching methodology, learning activities, and e-assessment methods, and it eases the teaching and learning process	100%	64%	71%	0	55%
Learning resources and student support	IN10. Learner support is offered according to the student’s profile and their specific needs	86%	50%	57%	0	18%
	IN09. The institution has procedures in place that cover learner support, including tutoring, pedagogical, technological, and administrative elements	79%	50%	64%	0	36%
	IN12. Hours of support are transparent and suit the needs of learners; for instance, periods of peak demand (evenings, weekends, holidays, etc.) are considered	79%	64%	71%	0	45%
	IN18. Learners receive guidelines/training in using MOOC resources (VLE, e-library, etc.)	71%	64%	64%	0	45%
	IN11. The learner support reflects characteristics of MOOC	50%	50%	50%	0	18%
Public information	IN27. The MOOC publishes information on completion rates, pass rates, and dropout rates	57%	50%	50%	0	18%
Ongoing monitoring and periodic review of programmes	IN30. MOOC are reviewed, updated, and improved	86%	64%	50%	0	55%
	IN29. The internal quality assurance system includes feedback to stakeholders (especially to learners)	79%	57%	57%	0	45%

## Discussion

This study characterises MOOCs and proposes a system of requirements and indicators for the evaluation, based on ENQA considerations. In this system, there are two key components. On the one hand, 11 requirements have been specified to define what a MOOC is and to rule out other types of training or courses that are not MOOCs. On the other hand, 23 indicators have been specified to be applied in the evaluation of the quality of any MOOC (on the understanding that it meets the above requirements).

The Delphi method applied was appropriate to the objective set and has enabled a consensus to be reached on the indicators for assessing the quality of MOOCs in e-learning. The first round of the method has discriminated which requirements and indicators should be included in the system and not too many proposals have been added, so that a good basis based on ENQA has been used; and in the second round 100% agreement has been reached.

Content indicators are included, allowing MOOC designers to assess the specificity of courses, one of the key aspects of course quality [23–25]. Indicators also allow the identified needs of MOOCs to be met, such as making them more interactive, considering the needs of learners and increasing the supply of MOOCs. Based on these results, shorter and more interactive MOOCs are recommended to encourage learners to enroll the courses.

The system of specific MOOC indicators resulting from this study improves the evaluation processes and quality of MOOCs present in the scientific literature [14, 18]. Its application would allow the minimum quality compliance demanded by the students of the courses [4] and evaluate the success of the MOOC through its results [15], homogenising and unifying criteria [18].

### Implications of the study

The outcome of this study could provide universities a guideline to identify what a MOOC (checklist) is and assess its quality, on the one hand, and will allow institutions to guarantee that the MOOCs offered are working properly. On the other hand, this system will allow future students of these courses to know which MOOCs are of the highest quality. The proposed system of the current study could have a preventive use evaluating *ex ante* the quality of the design of one MOOCs. It would be very useful to identify areas that can be improved in relation to the development of learning and evaluation processes in virtual environments. Many of the challenges that must be overcome in the creation and development of MOOCs, given their massive nature, could be solved after the application of the checklist of indicators.

### Limitations and future work

The main limitation of this study lies in the internationalisation of the sample and sample loss. Even though, it was found that there were no significant differences between the participating countries and the results are consistent. The evaluation of the implementation of the proposed system and the extension of the study to other forms of e-learning methods are proposed as possible studies.

## Supporting information

S1 File (PDF)
