# Supplementary material for: Quality criteria in MOOC: Comparative and proposed indicators
Source: PLoS One. 2022 Dec 5;17(12):e0278519. doi: 10.1371/journal.pone.0278519 (PMC9721481; doi:10.1371/journal.pone.0278519)
Supplement: S1 File — (PDF) [file pone.0278519.s001.pdf]

We request your valuable cooperation - this second round will not take you more than 5 minutes – in contributing your inclusion proposal (Personal opinion: Yes-1 or No-0) to the list of requirements and indicators. Please, reply to this message by attaching your EXCEL answer.

**REQUIREMENTS**	**RESULTS**	**PROPOSAL Inclusion (1/0)**	**PERSONAL OPINION A**	**B**	**C**	**D**	**E**	**F**	**G**	**H**	**I**	**J**	**K**
[RE11. The certification is optional]	100%	1	1	1	1	1	1	1	1	1	1	1	1
[RE01. The MOOC admission is massive (thousands) and is not limited]	93%	1	1	1	1	0	1	1	1	1	1	1	1
[RE03. The fee of the MOOC is free (there is no payment for registration)]	93%	1	1	1	1	1	1	1	1	1	1	1	1
[RE08. The main content media of the MOOC are multimedia, interactive (not textbook, pdf)]	86%	1	1	1	1	1	1	1	1	1	1	0	1
[RE09. The emphasis of the MOOC is focused on the learning process (not on evaluation)]	86%	1	1	1	1	1	1	1	1	1	1	1	1
[RE10. The MOOC e-assessment system is included (machine-scored; peer)]	79%	1	1	1	1	1	1	1	1	1	1	1	1
[RE04. The environment of the MOOC is open]	71%	1	1	1	1		1	1	1	1	1	1	0
[RE06. There is a variety and optional communication tools, including social networks]	71%	1	1	0	1	1	1	0	1	1	1	1	1
[RE02. There is no access requirement to the MOOC]	64%	0	1	0	1	0	1	0	1	1	0	1	0
[RE05. The educational staff contact and support are minimum or not expected]	64%	0	1	1	0	0	1	1	1	0	0	0	1
[RE07. The session is opened automatically]	50%	0	0	0	0	0	1	0	0	1	0	0	0

**INDICATORS**	**Relevance**	**Feseability**	**Comparability**	**PROPOSAL Inclusion (1/(**	**PERSONAL OPINION A**	**B**	**C**	**D**	**E**	**F**	**G**	**H**	**I**	**J**
IN05. Teaching methodologies and learning activities are chosen with the aim of achieving learning outcomes	100%	100%	86%	1	1	1	1	1	1	1	1	1	1	1
IN25. The MOOC considers ethical norms and government policy with respect to data protection and the privacy of learners	100%	86%	93%	1	1	1	1	1	1	1	1	1	1	1
IN06. Learning materials fit the pedagogical model and facilitate student learning	100%	86%	86%	1	1	1	1	1	1	1	1	1	1	1
IN21. Technological and pedagogical support services for educators are adequate, accessible, and timely	100%	79%	64%	1	1	1	1	1	1	1	1	1	1	1
IN22. The technical infrastructure ensures the accessibility of the MOOC programme by learners with special educational needs	100%	79%	86%	1	1	1	1	1	1	1	1	1	1	0
IN14. E-assessment methods are fit for purpose, allowing students to demonstrate the extent to which the intended learning outcomes have been achieved	100%	71%	71%	1	1	1	1	1	1	1	1	1	1	1
IN16. Learners are aware of plagiarism rules	100%	71%	86%	1	1	1	1	1	1	1	1	1	1	1
IN02. People involved in designing/developing/evaluating MOOC programmes have expertise in academic and technical aspects	93%	86%	71%	1	1	1	1	1	0	1	1	1	1	1
IN07. Learning materials are relevant and are reviewed and updated periodically.	93%	86%	64%	1	1	1	1	1	1	1	1	1	1	1
IN13. Learners are informed about the workload and pedagogical model of the MOOC programme	93%	79%	79%	1	1	1	1	1	1	1	1	1	1	0
IN15. Learners are clearly informed about the e-assessment	93%	79%	79%	1	1	1	1	1	1	1	1	1	1	1
IN26. The MOOC publishes reliable, complete, and up-to-date information on MOOC (i.e. recognition of qualifications, learning objectives, credits, requirements, assessment me	93%	71%	64%	1	1	1	1	1	1	1	1	1	1	1
IN17. Learners/prospective learners are informed about requirements concerning equipment, MOOC and digital skills, pre-knowledge and prerequisite subjects, and attendance	93%	71%	86%	1	1	0	1	1	1	0	1	1	1	1
IN08. The VLE provides the appropriate methods and tools that support effectively the achievement of the learning outcomes	93%	71%	79%	1	1	1	1	1	1	1	1	1	1	1
IN24. The MOOC guarantee the electronic security measures that guarantee standards of quality and information integrity and validity	86%	86%	79%	1	1	1	1	1	1	1	1	1	1	1
IN04. There are coordination mechanisms for the educational staff involved, if applicable	86%	86%	79%	1	1	1	1	1	1	1	1	1	1	1
IN03. Learner needs (including special educational needs if applicable) are considered when developing the learning model and the curricula design	86%	86%	71%	1	1	0	1	1	1	0	1	1	1	0
IN23. Technical requirements to enable the full and effective use of the system are clearly identified and published	86%	71%	86%	1	1	0	1	1	1	0	1	1	1	1
IN28. The MOOC foresees an evaluation system for improvement that includes satisfaction surveys of stakeholders, especially learners (quality assurance system of the course	86%	71%	57%	1	1	1	1	1	1	1	1	1	1	1
IN20. The technical infrastructure is aligned with the teaching methodology, learning activities, and e-assessment methods, and it eases the teaching and learning process	100%	64%	71%	0	1	0	0	1	1	0	1	1	1	0
IN19. ICT and pedagogy developments are analysed and implemented when appropriate	93%	64%	71%	0	1	1	0	1	1	1	1	1	1	1
IN30. MOOC are reviewed, updated, and improved	86%	64%	50%	0	0	0	0	1	1	0	0	1	1	1
IN10. Learner support is offered according to the student's profile and their specific needs	86%	50%	57%	0	0	0	0	1	0	0	0	1	0	0
IN12. Hours of support are transparent and suit the needs of learners; for instance, periods of peak demand (evenings, weekends, holidays, etc.) are considered	79%	64%	71%	0	1	0	0	1	1	0	1	1	0	0
IN31. Collected data is used in order to evaluate MOOC programmes (e.g. comparative analysis of course design)	79%	64%	50%	0	1	1	0	1	1	1	1	1	0	1
IN29. The internal quality assurance system includes feedback to stakeholders (especially to learners)	79%	57%	57%	0	1	0	0	1	1	0	1	1	0	0
IN09. The institution has procedures in place that cover learner support, including tutoring, pedagogical, technological, and administrative elements	79%	50%	64%	0	1	0	0	0	1	0	1	1	0	0
IN01. MOOC learning objectives is part of the overall strategy for the institution's development as well as the policy for quality assurance	71%	64%	57%	0	0	0	0	0	1	0	0	1	0	0
IN18. Learners receive guidelines/training in using MOOC resources (VLE, e-library, etc.)	71%	64%	64%	0	1	0	0	0	1	0	1	1	0	0
IN27. The MOOC publishes information on completion rates, pass rates, and dropout rates	57%	50%	50%	0	0	0	0	0	1	0	0	1	0	0
IN11. The learner support reflects characteristics of MOOC	50%	50%	50%	0	0	0	0	0	0	0	0	1	0	0

**NEW ITEMS PROPOSED BY EXPERTS**	**PERSONAL OPINION A**	**B**	**C**	**D**	**E**	**F**	**G**	**H**	**I**	**J**	**K**
IN32. The MOOC fosters interactions between learners.	1	1	1	0	0	1	1	1	1	1	1
IN33. Cultural factors are considered in the development of MOOC contents.	1	0	1	0	0	0	1	1	1	1	1
IN34. There are clear and defined roles for the educational staff involved in the MOOC, if applicable.	1	0	1	1	1	0	1	1	1	1	1
